# Prevalence and factors associated with mother and newborn skin-to-skin contact in Afghanistan

**DOI:** 10.1371/journal.pone.0324758

**Published:** 2025-05-21

**Authors:** Essa Tawfiq, Muhammad Haroon Stanikzai, Massoma Jafari, Zarghoon Tareen, Sayed Ali Shah Alawi, Zainab Ezadi, Abdul Wahed Wasiq, Omid Dadras

**Affiliations:** 1 The Kirby Institute, UNSW Sydney, Sydney, Australia; 2 Department of Public Health, Faculty of Medicine, Kandahar University, Kandahar, Afghanistan; 3 Department of Health Profession Education Research, University of Toronto, Ontario, Canada; 4 Department of Pediatrics, Faculty of Medicine, Kandahar University, Kandahar, Afghanistan; 5 UHI Project/JHPIEGO, Kabul, Afghanistan; 6 Master of Science in Midwifery, Reproductive Health, Kabul, Afghanistan; 7 Department of Internal Medicine, Faculty of Medicine, Kandahar University, Kandahar, Afghanistan; 8 Research Centre for Child Psychiatry, University of Turku, Turku, Finland; Jahangirnagar University, BANGLADESH

## Abstract

**Background:**

Mother-newborn skin-to-skin contact (SSC) involves placing the naked infant on the mother’s bare chest within the first hour of birth and is crucial for thermoregulation, bonding, breastfeeding initiation, and promoting neonatal health. This study examined the prevalence, and factors associated with SSC in Afghanistan.

**Methods:**

Data from the Afghanistan Multiple Indicator Cluster Survey (MICS) 2022–23 were used and analysed from ever-married women, aged 15–49 years, who delivered a live infant in the past 2 years. The outcome was SSC, placing the naked infant on the mother’s bare chest and initiating breastfeeding within the first hour of birth. Adjusted odds ratios [AOR: (95%CI)] of factors associated with SSC were obtained by a logistic regression model.

**Results:**

Of 11,992 women, 32.9% practiced SSC. The likelihood of SSC was greater in women with primary [1.38 (1.14–1.68)] and secondary or higher [1.29 (1.06–1.57)] education, in women who had access to media [1.36 (1.11–1.65)], and those who owned mobile phones [1.27 (1.11–1.45)]. The likelihood of SSC was lower in women who delivered at home [0.26 (0.21–0.33)], those who delivered at private clinics or hospitals [0.50 (0.41–0.61)], and those with cesarean section [0.12 (0.08–0.17)]. Women living in rural areas, and women with deliveries conducted by traditional birth attendants/community healthcare workers and by relatives/others had lower odds of SSC [0.76 (0.63–0.92), 0.37 (0.27–0.53), 0.45 (0.33–0.59), respectively].

**Conclusion:**

The low prevalence of SSC in Afghanistan highlights the need for targeted health interventions. Efforts should focus on improving access to public clinics and hospitals, enhancing education, training of healthcare providers, and leveraging media and mobile phone access to promote SSC. Interventions should prioritize rural women and women who have undergone cesarean sections to increase SSC rates and improve neonatal health outcomes.

## Introduction

The first 28 days of a newborn’s life are crucial, accounting for 47% of all deaths among children under five worldwide [1]. These deaths are often due to illnesses related to inadequate quality care at birth or the lack of skilled care and treatment immediately after birth and during the first few days [1]. Given this high risk of mortality, immediate interventions such as mother and newborn skin-to-skin contact (SSC) are vital for improving newborn health outcomes.

SSC involves placing the newborn naked (or in a diaper) on the mother’s bare chest [2]. The importance of SSC within the first hour of childbirth is to promote initiation of early breastfeeding, and the World Health Organization (WHO) recommends that SSC should be immediate after birth and be continued uninterrupted for at least 60 minutes to support mothers to initiate breastfeeding as soon as possible after birth [3]. This practice promotes early breastfeeding initiation, improves thermoregulation, and enhances neonatal survival while boosting maternal confidence [3–5]. SSC triggers vital reflexes in newborns, especially those born preterm or via cesarean section [3,5], and facilitates the delivery of colostrum, a highly nutritious first milk that contains essential antibodies and immune-boosting substances [3]. For mothers, SSC supports early placenta expulsion [6,7], reduces bleeding [7], lowers stress levels [8], and strengthens mother-infant bonding, which is mediated by increased oxytocin levels [9]. Additionally, SSC reduces the risk of neonatal hypoglycemia and the need for admission to intensive care units [10]. Recent WHO studies highlight that starting kangaroo mother care (KMC) with SSC and exclusive breastfeeding immediately after birth greatly improves survival rates of preterm or low-weight infants, and could prevent up to 150,000 infant deaths annually [11].

In Afghanistan, neonatal mortality remains alarmingly high, with 34 deaths per 1,000 live births, primarily due to prematurity (38%) and birth asphyxia (19%) [12]. Despite the proven benefits of SSC, its practice may be limited in Afghanistan, likely due to cultural and systemic barriers [13]. Cultural norms emphasizing modesty may deter mothers from engaging in SSC, as exposing the chest is deemed inappropriate [14,15]. This cultural pressure can cause SSC to be viewed as inappropriate or unacceptable, limiting its adoption [16]. The lack of privacy in healthcare facilities, coupled with overcrowded conditions, further limits SSC adoption [17]. Similarly, during home births, the presence of male relatives and lack of privacy inhibit mothers from practicing SSC [13,14].

Additionally, there may be limited awareness among healthcare providers and mothers about the importance of SSC for newborns, which hinders its widespread adoption [18]. Challenges within the healthcare system, such as limited resources, lack of training, and high patient-to-provider ratios, further complicate the implementation of SSC [18,19]. The 2016 Afghanistan National Maternal and Newborn Health Quality of Care Assessment reported that while 85% of newborns were dried immediately after birth, only 52% were placed in SSC, and just 62% of skilled birth attendants included thermal protection in essential newborn care [20]. This is particularly concerning as hypothermia is a major contributor to neonatal mortality [21].

Afghanistan is striving to meet the Sustainable Development Goal (SDG) target of reducing neonatal mortality to 12 deaths per 1,000 live births by 2030. To reach this goal, the country must substantially improve essential newborn care, including enhancing the practice of SSC. The latest Multiple Indicator Cluster Survey (MICS) 2022–23 reported that only 15.8% of newborns in Afghanistan receive SSC for thermal care [22]. This low prevalence underscores the urgent need to investigate the factors influencing SSC and re-evaluate its practice within the country.

Given Afghanistan’s ongoing socio-political instability, including the increasing humanitarian crises and forced repatriation of Afghan refugees from Pakistan and Iran [23,24], maternal and neonatal health attracts enormous attention because they are at the highest risk of morbidities and mortalities in such situations [25–27]. In this context, SSC within the first hour of delivery is an essential component of maternal and neonatal care [3]. However, research on SSC in Afghanistan remains limited. This study, therefore, seeks to examine the prevalence, and factors associated with SSC in the Afghan context.

## Methods

### Study design and data source

**Fig 1:**
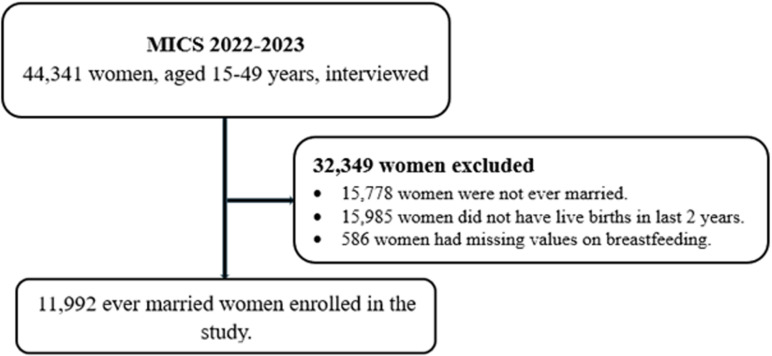
Study flowchart.

This cross-sectional study used data from the Afghanistan MICS 2022–2023, accessed on June 10, 2024 [22]. The MICS survey covers a wide range of indicators related to the situation of women and children, including child mortality, maternal and newborn health, and water and sanitation. Detailed descriptions of the survey design, sampling methods, and data collection are described elsewhere [22]. During the implementation of MICS, trained surveyors collected data from women of reproductive age (15–49 years old) [22]. In this study, we used and analysed data from 11,992 ever-married women who delivered a live infant in the past 2 years prior to the MICS survey (Fig 1).

### Study variables

The **outcome** was SSC, defined as placing the newborn on the mother’s bare chest and initiating breastfeeding within the first hour of birth. SSC was categorized as a binary outcome, coded as “yes” if the mother responded affirmatively to both of the following questions: whether the infant was placed on her bare chest after delivery, and whether breastfeeding was initiated within the first hour of birth. If the response to either question was “no,” the outcome was coded as “no.”

The **explanatory variables** were selected after a comprehensive literature review [10,28–33]. It includes maternal age (15–19, 20–24, 25–29, 30–39, 40–49 years), maternal education (no formal education, primary education, secondary/higher education), birth order (first child vs. second and more children), infant sex (male vs. female), household wealth status (lowest quintile up to highest quintile), residential area (urban vs. rural), place of delivery (public clinic/hospital, private clinic/hospital, and at home), type of delivery provider (doctor, midwife/nurse, traditional birth attendants (TBAs)/community healthcare worker (CHWs), relatives/other), cesarean section (“yes” if the delivery was by cesarean section, and “no” otherwise), small-size baby (“yes” if the size of the baby was reported to be very small by the interview mother, and “no” otherwise), antenatal care (ANC) visits (no visit, 1–3 visits, 4–7 visits, and ≥ 8 visits), woman used a mobile phone at least once a week in the last 3 months (yes/no), and access to media (yes/no). Access to media was defined as daily TV watching, radio listening, or newspaper reading (yes/no).

### Statistical analysis

Descriptive statistics were used to assess the distribution of sociodemographic characteristics among the study population. The chi-square test was used to examine the relationship between explanatory variables and SSC status. Univariate and multivariable logistic regression models were fitted and run to study the likelihood of SSC across the categories of explanatory variables. In the multivariable model, explanatory variables were selected based on the theoretical relevance after a thorough literature review [10,28–33], and the strength of their relationship with the outcome, was determined by the corresponding *p*-value. The *p*-values obtained from the univariate analysis were used to determine which explanatory variables to include in the multivariate model. Variables with a *p*-value of < 0.25 were added to the multivariate model. In the univariate analysis, women’s age and infant’s sex had *p*-values > 0.25; therefore, all other explanatory variables were added to the multivariate model except for these two variables. Odds ratio and 95% CI [OR (95%CI)] were obtained for the logistic regression analyses. All analyses accounted for sampling design and weight by defining the survey strata, primary sampling unit, and weight for all analyses in the study. The significant statistical level was set at *p* < 0.05. STATA version 18 was applied for data analyses [34].

### Ethical approval

The study was reviewed by the Research and Ethics Committee, Faculty of Medicine, Kandahar University, Afghanistan. The committee waived the ethical approval (Dated; 20/May/2024) because secondary data from the Multiple Indicator Cluster Survey (MICS) 2022–2023 were used and analyzed in this study.

## Results

As shown in Table 1, 32.9% of women practiced SSC. Of 11,992 women, over 6% were 15–19 years, and over 7% were 40–49 years of age. In terms of age, there was no statistical difference between women who practiced SSC and those who did not. However, statistically significant differences were observed between the groups of women in terms of education, birth order, wealth status, place of delivery, deliveries conducted by type of health provider, cesarean section, ANC visits, access to media, and mobile phone ownership. These differences were in favor of women who practiced SSC compared to women who did not. For example, the proportions of women with secondary or higher education were 18.7% vs. 10.9% for women who practiced SSC and for women who did not (details in Table 1).

**Table 1:** Baseline characteristics of ever-married women, by status of skin-to-skin contact.

Characteristics	Categories	Whether skin-to-skin contact was practiced			*p*-value
		Total n = 11,992	Yes n = 3,944 (32.9%)	No n = 8,048 (67.1%)	
Mothers’ age	15–19 years	6.1%	6.0%	6.1%	0.74
	20–24 years	24.8%	24.8%	24.9%	
	25–29 years	29.7%	30.3%	29.4%	
	30–39 years	32.3%	32.4%	32.3%	
	40–49 years	7.1%	6.6%	7.4%	
Mothers’ education	No formal education	75.9%	68.3%	80.1%	<0.001
	Primary	10.5%	13.1%	9.1%	
	Secondary/higher	13.6%	18.7%	10.9%	
Birth order	1^st^ child	16.0%	17.4%	15.3%	0.03
	2^nd^ or more children	84.0%	82.6%	84.7%	
Infant sex	Male	51.6%	51.6%	51.5%	0.94
	Female	48.4%	48.4%	48.5%	
Wealth status	Lowest quintile	20.7%	13.4%	24.7%	<0.001
	Second	20.9%	17.7%	22.7%	
	Middle	20.4%	20.1%	20.6%	
	Fourth	19.5%	23.7%	17.2%	
	Highest quintile	18.4%	25.2%	14.7%	
Residential area	Urban	23.2%	32.0%	18.4%	<0.001
	Rural	76.8%	68.0%	81.6%	
Place of delivery	Public clinic/hospital	56.0%	78.8%	43.6%	<0.001
	Home	33.5%	11.1%	45.7%	
	Private clinic/hospital	10.5%	10.2%	10.6%	
Type of delivery provider	Doctor	9.1%	12.3%	7.3%	<0.001
	Midwife/nurse	53.9%	73.0%	43.6%	
	TBAs/CHWs	11.8%	4.1%	16.0%	
	Relatives/other	25.2%	10.6%	33.2%	
Cesarean section	No	94.4%	97.9%	92.5%	<0.001
	Yes	5.6%	2.1%	7.5%	
Small-size baby	No	85.2%	86.2%	84.7%	0.21
	Yes	14.8%	13.8%	15.3%	
Antenatal care (ANC) visit	No visit	22.1%	15.4%	25.8%	<0.001
	1–3 visits	44.5%	45.4%	44.0%	
	4–7 visits	26.4%	30.7%	24.0%	
	≥8 visits	7.1%	8.5%	6.3%	
Access to mobile phone	No	63.1%	53.8%	68.2%	<0.001
	Yes	36.9%	46.2%	31.8%	
Access to media	No	80.0%	72.0%	84.0%	<0.001
	Yes	20.0%	28.0%	16.0%	

**Abbreviations:** TBAs, Traditional birth attendants; CHWs, Community healthcare workers.

Table 2 presents the likelihood of SSC from bivariate and multivariate analyses. Results from the multivariate analysis show that women’s education level was positively associated with SSC: women with primary education, and women with secondary or higher education had higher odds of practicing SSC, compared to women with no formal education [1.38 (1.14–1.68), 1.29 (1.06–1.57), respectively]. Similarly, women with access to media and women with ownership of mobile phones had higher odds of practicing SSC [1.36 (1.11–1.65), and 1.27 (1.11–1.45)], compared to women with no access to media and women with no ownership of mobile phones, respectively. The likelihood of SSC was lower in women who delivered at home [0.26 (0.21–0.33)], and in those delivered at private clinics or hospitals [0.50 (0.41–0.61)], compared to those delivered at public clinics or hospitals. Women who had cesarean section deliveries were less likely to practice SSC compared to those with vaginal deliveries [0.12 (0.08–0.17)]. Likewise, women living in rural areas, compared to those living in urban areas, women with deliveries conducted by TBAs/CHWs, and by relatives/other, compared to women whose deliveries were conducted by doctors, had lower odds of practicing SSC [0.76 (0.63–0.92, 0.37 (0.27–0.53), and 0.45 (0.33–0.59), respectively].

**Table 2:** Likelihood of skin-to-skin contact practiced by ever-married women.

Characteristics	Categories	Crude odds ratio (95% CI)	p-value	Adjusted odds ratio (95% CI)	p-value
Mothers’ age	15–19 years	Ref		Ref	
	20–24 years	1.02 (0.81–1.27)	0.89	–	–
	25–29 years	1.05 (0.84–1.31)	0.68	–	–
	30-39 years	1.02 (0.81–1.28)	0.87	–	–
	40–49 years	0.90 (0.68–1.20)	0.49	–	–
Mothers’ education	No formal education	Ref		Ref	
	Primary	1.69 (1.41–2.04)	<0.001	1.38 (1.14–1.68)	0.001
	Secondary/higher	2.02 (1.67–2.43)	<0.001	1.29 (1.06–1.57)	0.01
Birth order	1^st^ child	Ref		Ref	
	2^nd^ or more children	0.86 (0.75–0.99)	0.03	0.98 (0.84–1.14)	0.79
Infant sex	Male	Ref		Ref	
	Female	1.00 (0.91–1.09)	0.94	–	–
Wealth status	Lowest quintile	Ref		Ref	
	Second	1.44 (1.19–1.74)	<0.001	1.09 (0.90–1.33)	0.36
	Middle	1.80 (1.50–2.16)	<0.001	1.07 (0.89–1.30)	0.51
	Fourth	2.54 (2.06–3.13)	<0.001	1.15 (0.91–1.45)	0.24
	Highest quintile	3.16 (2.54–3.94)	<0.001	1.05 (0.79–1.40)	0.75
Residential area	Urban	Ref		Ref	
	Rural	0. 48 (0.41–0.56)	<0.001	0.76 (0.63–0.92)	0.006
Place of delivery	Public clinic/health post	Ref		Ref	
	Home	0. 13 (0.11–0.16)	<0.001	0.26 (0.21–0.33)	<0.001
	Private clinic/hospital	0.53 (0.43–0.65)	<0.001	0.50 (0.41–0.61)	<0.001
Type of delivery provider	Doctor	Ref		Ref	
	Midwife/nurse	1.00 (0.80–1.26)	0.97	0.87 (0.70–1.09)	0.22
	TBAs/CHWs	0.15 (0.11–0.22)	<0.001	0.37 (0.27–0.53)	<0.001
	Relatives/other	0.19 (0.15–0.24)	<0.001	0.45 (0.33–0.59)	<0.001
Cesarean section	No	Ref		Ref	
	Yes	0.27 (0.19–0.39)	<0.001	0. 12 (0.08–0.17)	<0.001
Small size baby	No	Ref		Ref	
	Yes	0. 89 (0.74–1.07)	0.21	0.94 (0.77–1.15)	0.56
Antenatal care (ANC) visits	No visit	Ref		Ref	
	1–3 visits	1.73 (1.49–2.02)	<0.001	0.97 (0.82-1.14)	0.68
	4–7 visits	2.15 (1.81–2.56)	<0.001	0.96 (0.80–1.15)	0.62
	≥8 visits	2.29 (1.47–2.97)	<0.001	1.06 (0.80–1.40)	0.71
Access to mobile phone	No	Ref		Ref	
	Yes	1.84 (1.63–2.07)	<0.001	1.27 (1.11–1.45)	<0.001
Access to media	No	Ref		Ref	
	Yes	1.96 (1.65–2.33)	<0.001	1.36 (1.11–1.65)	0.003

**Abbreviations:** TBA, Traditional birth attendants; CHW, Community healthcare workers.

## Discussion

This study found that only a third of Afghan women practiced SSC. The likelihood of SSC was higher in women with primary or higher education, and those with access to media and mobile phones. On the contrary, the likelihood of SSC was lower in women who delivered at home, those who delivered at private clinics or hospitals, women with deliveries conducted by TBAs/CHWs and by relatives/others, rural women, and those who had cesarean sections.

The SSC prevalence of 32.9% observed in this study is double that reported by the Afghanistan MICS 2022–23 [22]. This discrepancy may be attributed to differences in our calculation of SSC prevalence, as we excluded women who did not have a live birth in the past 2 years. The prevalence of SSC practice is lower than the 52% reported previously in Afghanistan [20]. Considering the importance of SSC within the first hour after childbirth and initiation of early breastfeeding, our definition of SSC covered the placement of the naked newborn on the mother’s bare chest and initiation of breastfeeding within 60 minutes of the delivery. Nevertheless, our prevalence aligns with findings from other low- and middle-income countries (LMICs), where SSC rates range from 28.0% to 45.7% [30,35,36]. The low SSC prevalence remains a pressing concern, emphasizing the need for targeted efforts to improve SSC practices in Afghanistan. The factors identified in our study should guide future interventions and policy developments.

This study showed that SSC prevalence was significantly higher among women with primary or higher education levels compared to those with no formal education. This is consistent with previous research in LMICs, where maternal education has been linked to a better understanding of neonatal care and improved SSC rates [30,35,37]. Our findings underscore the critical importance of women’s education, particularly in light of the recent restrictions on female education in Afghanistan [38]. The findings of this study reinforce the need to support continued advocacy for women’s education in Afghanistan. Additionally, public health campaigns aimed at raising awareness of SSC among less-educated women are urgently needed.

We found that women living in rural areas were less likely to practice SSC than those living in urban areas. This finding is consistent with the studies conducted in Ethiopia [36,39], and Nigeria [32]. Women living in rural areas tend to have poor access to maternal and child healthcare services, which could explain the observed association [32]. Moreover, earlier studies in Afghanistan [40–43], revealed that rural women are at a disadvantage in accessing maternal and child healthcare services. Therefore, it is essential to design interventions that specifically target this population. Policymakers must prioritize improving healthcare accessibility for rural mothers and newborns to address these inequalities.

Consistent with findings from other LMICs, our study found a positive association between deliveries at health facilities and SSC [28,30]. Access to maternal and neonatal healthcare in public health facilities can improve breastfeeding practices, including early initiation of breastfeeding [44], SSC [30], and exclusive breastfeeding [45]. Compared to private facilities, public healthcare facilities in Afghanistan are more closely aligned with national health policies and more frequently receive support and training from international partners to enhance maternal and newborn health services, including SSC practices [15,18,25,46]. In this study, we also observed that women whose deliveries were conducted by TBAs/CHWs and relatives/others were less likely to practice SSC. Therefore, increasing access to institutional deliveries may be a necessary policy consideration for improving SSC practices in Afghanistan [47]. Moreover, the findings highlight the need for targeted qualitative improvement initiatives, continuous training of healthcare workers, and public awareness efforts to ensure consistent SSC practices in Afghanistan. In addition to education for healthcare workers, public awareness campaigns may help to educate TBA/CHWs and relatives/others about the importance of SSC which may contribute to consistent SSC practices in Afghanistan.

Another important finding is the negative association of cesarean sections with SSC practices in our study, which corroborates with findings from previous studies [28,30]. A plausible reason could be the delay in initiating breastfeeding due to the recovery of the mother post-cesarean section operation. Additionally, the condition of the newborn after a cesarean section can also impact SSC practices [28]. Newborns delivered via cesarean section may experience distress or other complications, such as respiratory issues, which can necessitate immediate medical attention and further delay SSC [28,35]. This finding suggests that targeted interventions to improve SSC for newborns delivered by cesarean section are needed at health facilities, given WHO emphasizes supporting the dyad to engage in SSC practice, regardless of the mode of delivery [3]. The evidence from a literature review suggests that with appropriate collaboration of healthcare workers in maternity wards, SSC during cesarean section surgery can be effectively implemented [48]. To improve SSC after cesarean sections, healthcare facilities can assign a dedicated staff member to monitor SSC, ensuring newborn safety and supporting maternal bonding [49]. Staff education on SSC benefits—such as promoting breastfeeding, calming the newborn, and reducing stress—can strengthen team commitment [50]. Adjusting operating room protocols, like positioning the newborn on the mother’s chest with warm blankets, can also facilitate SSC [49,50]. Institutional policies should make SSC a standard practice to minimize mother-infant separation, aligning with WHO and UNICEF recommendations [51].

Data from LMICs consistently highlight the strong association between maternal mobile ownership and improved newborn care, such as better child feeding and timely immunization [52,53]. Similarly, we found that women with access to mobile phones were 1.2 times as likely to practice SSC compared to those with no access to mobile phones. Access to mobile phones likely enhances exposure to health education on breastfeeding and maternal and child health, as seen in other LMICs [53,54]. Recent evidence from Afghanistan identifies mobile health (mHealth) technologies as a potential opportunity to promote maternal and newborn health [55,56]. Thus, mHealth interventions may potentially enhance SSC practices, and may also contribute to improving other maternal healthcare utilization and newborn care indicators in the country.

Finally, consistent with studies from sub-Saharan Africa [28] and Nigeria [57], mothers’ access to media was strongly associated with SSC practice; e.g., women with access to media were about 1.3 times as likely to practice SSC than those with no access to media. Media plays an essential role in disseminating information on newborn care, particularly to less-educated women [58,59]. Educational campaigns through various media platforms have proven effective in improving maternal healthcare utilization and SSC practices in LMICs [58,60]. Therefore, expanding media-based education on maternal and child health in Afghanistan should be prioritized.

### Limitations

This study has some limitations. First, recall bias may have affected the accuracy of the data, as women were asked to report events that took place several months ago. Moreover, there were slightly over 1% (131 observations) with responses of “don’t know” for the SSC outcome, and this percent of responses were coded as “no SSC” for the outcome. This could have led to either under or over-reported SSC practice. Second, the MICS did not collect data on mother’s knowledge of breastfeeding; thus, we couldn’t examine the association between this important predictor and SSC practice. Furthermore, we were unable to account for other important predictors of SSC practice such as healthcare provider training and attitudes, prenatal breastfeeding intention, counseling on breastfeeding during ANC visits, cultural and social norms, maternal mental health, support from family or birth attendants, and previous birth experience. Hence, further research is warranted to examine them.

## Conclusion

Health interventions should be designed and implemented to address the low prevalence of SSC in Afghanistan. These interventions should focus on improving access to public healthcare facilities, enhancing educational opportunities for women, training healthcare providers, and promoting the use of media and mobile phones for health education. Special attention should be given to rural women and women who undergo cesarean sections. Prioritizing these groups is essential to increasing SSC rates and improving maternal and neonatal health outcomes in Afghanistan.

## Supporting information

S1 File Human_Subjects_Research_Checklist. (PDF)

## References

[1] World Health Organization. Newborn mortality 2024. Available from: https://www.who.int/news-room/fact-sheets/detail/newborn-mortality.

[2] Kostandy RR, Ludington-Hoe SM. The evolution of the science of kangaroo (mother) care (skin-to-skin contact). Birth Defects Res. 2019;111(15):1032–43. doi: 10.1002/bdr2.1565 31419082

[3] WHO and UNICEF. Protecting, promoting and supporting breastfeeding in facilities providing maternity and newborn services: implementing the revised Baby-friendly Hospital Initiative. 2018. ISBN 978-92-4-151380-7.

[4] Huang J-Z, Chen C-N, Lee C-P, Kao C-H, Hsu H-C, Chou A-K. Evaluation of the Effects of Skin-to-Skin Contact on Newborn Sucking, and Breastfeeding Abilities: A Quasi-Experimental Study Design. Nutrients. 2022;14(9):1846. doi: 10.3390/nu14091846 35565813 PMC9101996

[5] Smith ER, Hurt L, Chowdhury R, Sinha B, Fawzi W, Edmond KM, et al. Delayed breastfeeding initiation and infant survival: A systematic review and meta-analysis. PLoS One. 2017;12(7):e0180722. doi: 10.1371/journal.pone.0180722 28746353 PMC5528898

[6] Marín Gabriel MA, Llana Martín I, López Escobar A, Fernández Villalba E, Romero Blanco I, Touza Pol P. Randomized controlled trial of early skin-to-skin contact: effects on the mother and the newborn. Acta Paediatr. 2010;99(11):1630–4. doi: 10.1111/j.1651-2227.2009.01597.x 19912138

[7] Essa RM, Ismail N. Effect of early maternal/newborn skin-to-skin contact after birth on the duration of third stage of labor and initiation of breastfeeding. J Nurs Educ Pract. 2015;5(4):98.

[8] Handlin L, Jonas W, Petersson M, Ejdebäck M, Ransjö-Arvidson A-B, Nissen E, et al. Effects of sucking and skin-to-skin contact on maternal ACTH and cortisol levels during the second day postpartum-influence of epidural analgesia and oxytocin in the perinatal period. Breastfeed Med. 2009;4(4):207–20. doi: 10.1089/bfm.2009.0001 19731998

[9] Winberg J. Mother and newborn baby: mutual regulation of physiology and behavior--a selective review. Dev Psychobiol. 2005;47(3):217–29. doi: 10.1002/dev.20094 16252290

[10] Lord LG, Harding JE, Crowther CA, Lin L. Skin-to-skin contact for the prevention of neonatal hypoglycaemia: a systematic review and meta-analysis. BMC Pregnancy Childbirth. 2023;23(1):744. doi: 10.1186/s12884-023-06057-8 37865757 PMC10590034

[11] World Health Organization. Immediate kangaroo mother care post-birth critical for saving lives, new research shows. 2023. https://www.unicef.org.uk/babyfriendly/kangaroo-care-research/.

[12] UNICEF. countdown to 2030 country Profile: Afghanistan. 2024. Available from: https://data.unicef.org/countdown-2030/country/Afghanistan/1/.

[13] Lamberti-Castronuovo A, Valente M, Bocchini F, Trentin M, Paschetto M, Bahdori GA, et al. Exploring barriers to access to care following the 2021 socio-political changes in Afghanistan: a qualitative study. Confl Health. 2024;18(1):36. doi: 10.1186/s13031-024-00595-4 38658962 PMC11044283

[14] Najafizada SAM, Bourgeault IL, Labonté R. Social determinants of maternal health in Afghanistan: a review. Cent Asian J Glob Health. 2017;6(1):240. 29138735 10.5195/cajgh.2017.240 PMC5675389

[15] Arnold R, van Teijlingen E, Ryan K, Holloway I. Understanding Afghan healthcare providers: a qualitative study of the culture of care in a Kabul maternity hospital. BJOG. 2015;122(2):260–7. doi: 10.1111/1471-0528.13179 25394518 PMC4489341

[16] Lydon MM, Maruf F, Tappis H. Facility-level determinants of quality routine intrapartum care in Afghanistan. BMC Pregnancy Childbirth. 2021;21(1):438. doi: 10.1186/s12884-021-03916-0 34162347 PMC8223289

[17] Thommesen T, Kismul H, Kaplan I, Safi K, Van den Bergh G. “The midwife helped me. otherwise I could have died”: women’s experience of professional midwifery services in rural Afghanistan - a qualitative study in the provinces Kunar and Laghman. BMC Pregnancy Childbirth. 2020;20(1):140. doi: 10.1186/s12884-020-2818-1 32138695 PMC7059669

[18] Atiqzai F, Manalai P, Amin SS, Edmond KM, Naziri M, Soroush MS, et al. Quality of essential newborn care and neonatal resuscitation at health facilities in Afghanistan: a cross-sectional assessment. BMJ Open. 2019;9(8):e030496. doi: 10.1136/bmjopen-2019-030496 31473621 PMC6720229

[19] Naziri M, Higgins-Steele A, Anwari Z, Yousufi K, Fossand K, Amin SS, et al. Scaling up newborn care in Afghanistan: opportunities and challenges for the health sector. Health Policy Plan. 2018;33(2):271–82. doi: 10.1093/heapol/czx136 29190374

[20] UNICEF. Afghanistan National Maternal and Newborn Health Quality of Care Assessment 2016. 2016. Available from: www.unicef.org/afghanistan/media/1806/file/afg-report-MNH-QoC2016.pdf.

[21] Abdul-Mumin A, Boi-Dsane NAA, Oladokun ST, Owusu SA, Ansah P. A retrospective data analysis on prevalence and risk factors for hypothermia among sick neonates at presentation to the neonatal intensive care unit of the Tamale Teaching Hospital. PLoS One. 2024;19(5):e0303159. doi: 10.1371/journal.pone.0303159 38753864 PMC11098396

[22] Afghanistan multiple indicator cluster survey (MICS), 2022-2023. https://www.unicef.org/afghanistan/reports/afghanistan-multiple-indicator-cluster-survey-mics-2022-2023

[23] Jehangir A. Finding peace journalism: An analysis of Pakistani media discourse on Afghan refugees and their forced repatriation from Pakistan. Media War Conflict. 2023;16(4):582–98. doi: 10.1177/17506352221149559

[24] Ruhani A, Keshavarzi S, Kızık B, Çakal H. Formation of hatred emotions toward Afghan refugees in Iran: A grounded theory study. Peace and Conflict: J Peace Psychol. 2023;29(4):355–64. doi: 10.1037/pac0000685

[25] Glass N, Jalalzai R, Spiegel P, Rubenstein L. The crisis of maternal and child health in Afghanistan. Confl Health. 2023;17(1):28. doi: 10.1186/s13031-023-00522-z 37308945 PMC10262474

[26] Stanikzai M, Wafa M, Rahimi B, Sayam H. Conducting health research in the current Afghan society: challenges, opportunities, and recommendations. Risk Manag Healthc Policy. 2023;16:2479–83. 38024503 10.2147/RMHP.S441105 PMC10662640

[27] Dadras O, Stanikzai MH, Jafari M, Tawfiq E. Early childhood development and its associated factors among children aged 36-59 months in Afghanistan: evidence from the national survey 2022-2023. BMC Pediatr. 2024;24(1):734. doi: 10.1186/s12887-024-05222-y 39538223 PMC11562857

[28] Aboagye RG, Boah M, Okyere J, Ahinkorah BO, Seidu A-A, Ameyaw EK, et al. Mother and newborn skin-to-skin contact in sub-Saharan Africa: prevalence and predictors. BMJ Glob Health. 2022;7(3):e007731. doi: 10.1136/bmjgh-2021-007731 35296462 PMC8928283

[29] Aboagye RG, Okyere J, Dowou RK, Adzigbli LA, Tackie V, Ahinkorah BO, et al. Prevalence and predictors of mother and newborn skin-to-skin contact at birth in Papua New Guinea. BMJ Open. 2022;12(9):e062422. doi: 10.1136/bmjopen-2022-062422 36691147 PMC9445788

[30] Ali NB, Priyanka SS, Bhui BR, Herrera S, Azad MR, Karim A, et al. Prevalence and factors associated with skin-to-skin contact (SSC) practice: findings from a population-based cross-sectional survey in 10 selected districts of Bangladesh. BMC Pregnancy Childbirth. 2021;21(1):709. doi: 10.1186/s12884-021-04189-3 34686143 PMC8532372

[31] Daniels F, Sawangkum A, Kumar A, Coombs K, Louis-Jacques A, Ho TTB. Skin to Skin Contact Correlated with Improved Production and Consumption of Mother’s Own Milk. Breastfeed Med. 2023;18(6):483–8. doi: 10.1089/bfm.2022.0297 37335327 PMC10282785

[32] Ekholuenetale M, Barrow A, Arora A. Skin-to-skin contact and breastfeeding practices in Nigeria: a study of socioeconomic inequalities. Int Breastfeed J. 2022;17(1):2. doi: 10.1186/s13006-021-00444-7 34980169 PMC8725355

[33] Kristoffersen L, Bergseng H, Engesland H, Bagstevold A, Aker K, Støen R. Skin-to-skin contact in the delivery room for very preterm infants: a randomised clinical trial. BMJ Paediatr Open. 2023;7(1):e001831. doi: 10.1136/bmjpo-2022-001831 36958792 PMC10039990

[34] StataCorp. Stata Statistical Software: Release 18. College Station, TX: StataCorp LLC. 2023.

[35] Aboagye RG, Ahinkorah BO, Seidu A-A, Anin SK, Frimpong JB, Hagan JE Jr. Mother and newborn skin-to-skin contact and timely initiation of breastfeeding in sub-Saharan Africa. PLoS One. 2023;18(1):e0280053. doi: 10.1371/journal.pone.0280053 36626377 PMC9831337

[36] Mose A, Adane D, Abebe H. Skin-to-Skin Care Practice and Its Associated Factors Among Postpartum Mothers in Gurage Zone, Southern Ethiopia: A Cross-Sectional Study. Pediatric Health Med Ther. 2021;12:289–97. doi: 10.2147/PHMT.S306411 34163284 PMC8216067

[37] Debella A, Mussa I, Getachew T, Eyeberu A. Level of skin-to-skin care practices among postnatal mothers in Ethiopia. A systematic review and meta-analysis. Heliyon. 2024;10(8):e29732. doi: 10.1016/j.heliyon.2024.e29732 38665590 PMC11044043

[38] Ahmad A, Haqmal M. The Taliban’s ban on Afghan women attending university is eroding hopes for the future. BMJ. 2023;380:653. doi: 10.1136/bmj.p653 36944426

[39] Girma D, Abita Z. Rural versus urban variations of factors associated with early initiation of breastfeeding in Ethiopia. Heliyon. 2024;10(13):e33427. doi: 10.1016/j.heliyon.2024.e33427 39027529 PMC467058

[40] Stanikzai MH, Tawfiq E, Suwanbamrung C, Wasiq AW, Wongrith P. Predictors of antenatal care services utilization by pregnant women in Afghanistan: Evidence from the Afghanistan Health Survey 2018. PLoS One. 2024;19(10):e0309300. doi: 10.1371/journal.pone.0309300 39356654 PMC11446418

[41] Malik MA, Sinha R, Priya A, Rahman MHU. Barriers to healthcare utilization among married women in Afghanistan: the role of asset ownership and women’s autonomy. BMC Public Health. 2024;24(1):613. 38408956 10.1186/s12889-024-18091-y PMC10898116

[42] Tawfiq E, Azimi MD, Feroz A, Hadad AS, Soroush MS, Jafari M, et al. Predicting maternal healthcare seeking behaviour in Afghanistan: exploring sociodemographic factors and women’s knowledge of severity of illness. BMC Pregnancy Childbirth. 2023;23(1):561. doi: 10.1186/s12884-023-05750-y 37533023 PMC10398983

[43] Dadras O, Suwanbamrung C, Jafari M, Stanikzai MH. Prevalence of stunting and its correlates among children under 5 in Afghanistan: the potential impact of basic and full vaccination. BMC Pediatr. 2024;24(1):436. doi: 10.1186/s12887-024-04913-w 38971723 PMC11227132

[44] Woldeamanuel BT. Trends and factors associated to early initiation of breastfeeding, exclusive breastfeeding and duration of breastfeeding in Ethiopia: evidence from the Ethiopia Demographic and Health Survey 2016. Int Breastfeed J. 2020;15(1):3. doi: 10.1186/s13006-019-0248-3 31924229 PMC6953467

[45] Awoke S, Mulatu B. Determinants of exclusive breastfeeding practice among mothers in Sheka Zone, Southwest Ethiopia: A cross-sectional study. Public Health Pract (Oxf). 2021;2:100108. doi: 10.1016/j.puhip.2021.100108 36101636 PMC9461297

[46] Rahimzai M, Amiri M, Burhani NH, Leatherman S, Hiltebeitel S, Rahmanzai AJ. Afghanistan’s national strategy for improving quality in health care. Int J Qual Health Care. 2013;25(3):270–6. doi: 10.1093/intqhc/mzt013 23485422 PMC3671737

[47] International MotherBaby Childbirth Organization. The international childbirth initiative 2018. https://icichildbirth.org/initiative/. 2018.

[48] Stevens J, Schmied V, Burns E, Dahlen H. Immediate or early skin-to-skin contact after a Caesarean section: a review of the literature. Matern Child Nutr. 2014;10(4):456–73. doi: 10.1111/mcn.12128 24720501 PMC6860199

[49] Stevens J, Schmied V, Burns E, Dahlen HG. Who owns the baby? A video ethnography of skin-to-skin contact after a caesarean section. Women Birth. 2018;31(6):453–62. doi: 10.1016/j.wombi.2018.02.005 29496385

[50] Deys L, Wilson PV, Meedya DS. What are women’s experiences of immediate skin-to-skin contact at caesarean section birth? An integrative literature review. Midwifery. 2021;101:103063. doi: 10.1016/j.midw.2021.103063 34157585

[51] WHO/UNICEF. Advocacy brief: breastfeeding in emergency situations. 2018. Available from: https://www.globalbreastfeedingcollective.org/media/376/file

[52] Pesando LM, Qiyomiddin K. Mobile phones and infant health at birth. PLoS One. 2023;18(9):e0288089. doi: 10.1371/journal.pone.0288089 37708229 PMC10501678

[53] Knop MR, Nagashima-Hayashi M, Lin R, Saing CH, Ung M, Oy S, et al. Impact of mHealth interventions on maternal, newborn, and child health from conception to 24 months postpartum in low- and middle-income countries: a systematic review. BMC Medicine. 2024;22(1):196. 38750486 10.1186/s12916-024-03417-9 PMC11095039

[54] De P, Pradhan MR. Effectiveness of mobile technology and utilization of maternal and neonatal healthcare in low and middle-income countries (LMICs): a systematic review. BMC Womens Health. 2023;23(1):664. doi: 10.1186/s12905-023-02825-y 38082424 PMC10714653

[55] Lebrun V, Dulli L, Alami SO, Sidiqi A, Sultani AS, Rastagar SH, et al. Feasibility and Acceptability of an Adapted Mobile Phone Message Program and Changes in Maternal and Newborn Health Knowledge in Four Provinces of Afghanistan: Single-Group Pre-Post Assessment Study. JMIR Mhealth Uhealth. 2020;8(7):e17535. doi: 10.2196/17535 32706690 PMC7399960

[56] Noh J, Im Y, Kim K, Kim M, Kwon Y, Cha J. Digital and economic determinants of healthcare in the crisis-affected population in Afghanistan: access to mobile phone and socioeconomic barriers. Healthcare. 2021;9(5). 10.3390/healthcare9050506 PMC8145486 33925698

[57] Ekholuenetale M, Barrow A, Benebo FO, Idebolo AF. Coverage and factors associated with mother and newborn skin-to-skin contact in Nigeria: a multilevel analysis. BMC Pregnancy Childbirth. 2021;21(1):603. doi: 10.1186/s12884-021-04079-8 34481455 PMC8418713

[58] Aboagye RG, Seidu A-A, Ahinkorah BO, Cadri A, Frimpong JB, Hagan JE, et al. Association between frequency of mass media exposure and maternal health care service utilization among women in sub-Saharan Africa: Implications for tailored health communication and education. PLoS One. 2022;17(9):e0275202. doi: 10.1371/journal.pone.0275202 36174071 PMC9522280

[59] Tawfiq E, Stanikzai MH, Tareen Z, Alawi SAS, Wasiq AW, & Dadras O. Factors influencing early initiation of breastfeeding in Afghanistan: secondary analysis of the Afghanistan MICS 2022-23. International Breastfeeding J. 2025;20(1):30. 10.1186/s13006-025-00723-7 PMC11987306 40217460

[60] Sharma S, Adhikari B, Pandey AR, Karki S, K C SP, Joshi D, et al. Association between media exposure and maternal health service use in Nepal: A further analysis of Nepal Demographic and Health Survey-2022. PLoS One. 2024;19(3):e0297418. doi: 10.1371/journal.pone.0297418 38466757 PMC10927118

