# Supplementary material for: Prevalence and factors associated with mother and newborn skin-to-skin contact in Afghanistan
Source: PLoS One. 2025 May 21;20(5):e0324758. doi: 10.1371/journal.pone.0324758 (PMC12094735; doi:10.1371/journal.pone.0324758)
Supplement: Attachment — Submitted filename: Response Letter.pdf [file pone.0324758.s003.pdf]

Dear Respected Editor,

We would like to thank you and the esteemed reviewers for the thoughtful evaluation of our manuscript (ID Number, **PONE-D-24-41027**) entitled ***"Prevalence and factors associated with mother and newborn skin-to-skin contact in Afghanistan"***. Please find our revised manuscript (with highlighted changes), which we believe is substantially strengthened now that we have incorporated reviewers' recommendations.

Journal requirements

1. Please ensure that your manuscript meets PLOS ONE's style requirements, including those for file naming.

Response: Thank you. We have made the manuscript including tables, figures, and references as per journal's style requirements.

2. Your ethics statement should only appear in the Methods section of your manuscript. If your ethics statement is written in any section besides the Methods, please delete it from any other section.

Response: Thank you. We have made corrections.

Response to Reviewers' comments

Responses to Reviewer 1 comments

Reviewer #1: Skin-to-skin care (SSC), also known as kangaroo care, is a well-established evidence-based practice that has been shown to improve neonatal outcomes. This study investigates the prevalence and barriers to SSC implementation. The findings are significant and support the continued promotion of SSC, the highlighted interventions are reasonable and feasible.

Response: We thank you for the thoughtful comments and review.

1. Additional references are needed to strengthen the evidence base (references are needed in lines 86, 91, 200, and 249).

Response: Thank you so much for your meticulous observation. We have added relevant citations **(Please see lines 86, 91, 231, and 271).**

2. Lines 211-212 are not needed here, already mentioned more than once in the manuscript. Please delete.

Response: Thank you for this suggestion. In our revised analysis, age was not significant. Hence, we deleted the suggested sentences along with the mention paragraph.

3. The discussion section is redundant, and could be condensed to focus on the key findings and implications.

Response: Thank you so much. We agree that the discussion section is redundant. Moreover, two other variables were significant in revised analyses, which required further discussion. Other reviewers have also requested elaboration on some points in discussion. We tried to have condense the discussion and hope will be acceptable to the respected reviewer.

4. Were there any differences in the likelihood of SSC based on the gender of the delivery provider (physician, nurse, midwife, etc.)?

Response: Thank you so much for bringing this to our attention. The gender of delivery provider was not in the survey questionnaire and not reported by the survey participants; however, the type of delivery provider was reported. A type of delivery provider is midwives who are mainly female health workers in Afghanistan. In the revised multivariate results, the likelihood of SSC by women whose delivery was conducted by midwife/nurse is 0.87(0.70-1.09), compared to the deliveries conducted by doctors. Of note is the survey questionnaire did not collect data separately for midwives and for nurses who conducted the deliveries. It is most likely that the midwives/nurses who conducted deliveries were female health workers **(Please see Tables 1 and 2).**

5. Did mothers who received breastfeeding counseling during pregnancy have a different likelihood of engaging in SSC?

Response: Thank you for this suggestion. We agree as counseling during pregnancy is an important determinant of SSC. However, the MICS data did not collect information on breastfeeding counseling for mothers during pregnancy (during ANC visits). Nonetheless, we have added this to the limitation section of the study **(Please see lines 309-310 on Page 16).**

Responses to Reviewer 2 comments

Reviewer #2: Thank you for the opportunity to peer review this important research regarding skin-to-skin contact and early breastfeeding initiation. The manuscript is written in a concise manner with strong supporting rationale. I would like to offer some suggestions for consideration:

Response: Thank you for the detailed review and thoughtful feedback.

1. Line 115- Can you add some details about the MICS such as number of questions and type of questions (i.e. multiple choice, how it was administered)? In which languages was the survey available? Etc.

Response: Thank you for this comment. MICS is a survey similar to Demographic Health Survey, and the survey questionnaire includes different sections related to child health and nutrition, vaccination, growth and development, and related to maternal health (e.g., ANC, PNC, and childbirth). We have provided the citation for details of the survey design, sampling methods, and data collection **(Please see lines 114-117 on Page 5)**.

2. Line 117- suggest use the word "live infant" instead of "child".

Response: Thank you for this comment. We agree and we have revised throughout the manuscript **(Please see line 119 on Page 5).**

3. Line 126 - Suggest to analyze adolescents separately (19 years of age and less). This will make the groups more balanced and helpful to identify targeted education interventions. I see in Table TM 4.2 they have broken down age categories, and list as "less than 20" years of age (p. 75) of the MICS survey.

Response: Thank you for this suggestion. Thank you for this suggestion. We have revised the results based on your advice **(Please see Tables 1 and 2).**

4. Line 130 - suggest spelling out cesarean instead of c-section. Also suggest rewording the sentence "("yes" if the delivery was by cesarean…"

Response: Thank you for your meticulous observation. We have replaced the C-section with cesarean section throughout the manuscript. We have also corrected the suggested sentence **(Please see line 135 on Page 6).**.

5. Line 131-132- the "small-size baby" category seems highly subjective. Was there any data on birth weight? Can you report SGA or LBW instead?

Response: Thank you for this comment. We agree that small-size baby seems highly subjective. In the survey questionnaire and in the MICS dataset, there is a question on the weight of newborn (in gram); however, there are nearly 80% missing values on the

variable. Therefore, it is not possible to report to SGA or LBW.

6. Line 133- What's the reference for these categories of antenatal care visits? Four visits would not be adequate prenatal care as defined by other authors. There is certainly lots of debate about what is considered to be adequate. See recent integrative review https://onlinelibrary.wiley.com/doi/10.1111/jmwh.13459
   In the Unicef data summary, WHO is cited, with a minimum of 8 antenatal care visits recommended. (p. 72) https://mics.unicef.org/sites/mics/files/Afghanistan%202022-23%20MICS_English.pdf. Suggest reanalyzing the data including the following categories of number of antenatal visits: 4-7, 8 or more.

Response: Thank you for this suggestion. We have reanalyzed the data, considering your suggestion **(Please see Tables 1 and 2).**

7. Line 138- correction – it is chi-square

Response: Thank you for your meticulous observation. We have corrected **(Please see line 144 on Page 6).**

8. Line 146- add citation for STATA. What variables did you use when running the adjusted analysis? You need to add this.

Response: Thank you for this suggestion. STATA citation added **(Please see line 156 on Page 7)**. We have described that we included those explanatory variables which provided a p-value <0.25 in the univariate regression analysis **(Please see lines 148-152 on Page 7).**

9. Line 156- add the word respectively to the end of the sentence ending in SSC.

Response: Thank you for noticing this. We have rephrased this paragraph **(Please see line on 164 Page 7).**

10. Line 168 - will need to recalculate if using different age group categories as previously suggested.

Response: Thank you. We have recalculated using the suggested categories **(Please see Tables 1 and 2).**

11. Line 176- suggest “vaginal” instead of normal deliveries.

Response: Thank you for this comment. We have replaced the normal deliveries with vaginal deliveries **(Please see line 183 on Page 8)**.

12. Table 1. Suggest including standardized differences with a standardized difference of 0.10 or greater indicating an imbalance in groups (instead of p-values). Include a legend at the bottom of the table or spell out ANC in table.

Response: Thank you for this comment. We have used the sample weights from MICS as per your recommendation, and revised results in Table1, using chi square test. With respect to using a standardized difference of 0.10 or greater, we would kindly request the reviewer to accept our chi-square analyses because (i) determining the threshold for the difference is subjective (e.g., 0.10 or greater), and (ii) the use of chi square test for categorical variables is a well-established approach, with the corresponding p-values to indicate the level of statistical significance.

13. Line 196-197. Suggest restating the conclusion for women from higher socioeconomic backgrounds. The second quintile is not much higher than the first quintile. The other quintiles were non-significantly associated in the adjusted analysis.

Response: Thank you so much. After adding sampling weight to the data analysis as suggested by reviewers and MICS guideline, wealth was not associated with SSC practice. Hence, we deleted the suggested paragraph.

14. Line 202 – Re inclusion criteria of live birth within the last 2 years. Does this finding suggest that there may be an increasing trend in the use of SSC? Have there been any policy changes or public health campaigns that might have contributed to this? Certainly you reported that public clinics/hospitals were supporting this practice more so than private clinics/hospitals. Suggest to elaborate.

Response: Thank you for this comment. Actually, the inclusion criteria are similar with SSC definition described in MICS survey. In MICS 2022-2023 survey, the have also used the number of women with no live births in the denominator, which is an error in the survey report. We have now correctly reported the SSC prevalence in Afghanistan. Previously, a higher prevalence was reported **(Please see line 233 on Page 12)**. We have added relevant citation. We have also concluded that the low prevalence requires urgent interventions **(Please see line 235-237 on Page 12)**. For public clinics to practice SSC more often, we have described a possible rationale with relevant citations **(Please see lines 257-260 on Page 13)**.

15. Line 219- Suggest the following sentence "The findings of this study reinforce the need to support continued advocacy for women's education in Afghanistan".

Response: Thank you so much for this suggestion. We have revised the sentence **(Please see lines 243-244 on Page 12)**.

16. Line 222- Suggest "Consistent" instead of "in line with…".

Response: Thank you for this suggestion. This paragraph has been removed from the discussion as wealth status was not associated with SSC practices.

17. Line 224-225- Would this also be related to receiving health education about the importance of SSC and early breastfeeding initiation?

Response: Thank you. This paragraph has been removed from the discussion as wealth status was not associated with SSC practices.

18. Line 245- Suggest to include citation to the International Childbirth Initiative: 12 Steps to Safe and Respectful Mother-Baby Family Maternity Care. https://icichildbirth.org/initiative/

Response: Thank you for bringing this to our attention. We have added this very relevant citation **(Please see line 263 on Page 13).**

19. Line 253- Suggest "WHO emphasizes supporting the dyad to engage in SSC…"

Response: Thank you for this suggestion. We have revised the sentence **(Please see lines 274-275 on Page 14)**.

20. Line 256- Suggest to describe your recommendation in more detail. Often transient tachypnea of the newborn can be resolved by placing the newborn on the mother's chest, with close observation of the newborn. Perhaps this is an education component for staff. Someone on the team needs to be assigned to the baby for safety reasons (to ensure safe postnatal adaptation). Can also cite the Global Breastfeeding Collective Advocacy Brief. https://www.globalbreastfeedingcollective.org/media/376/file/Breastfeeding%20in%20emergency%20situations.pdf

Response: Thank you for this suggestion. We have added further details into the revised version of the manuscript **(Please see lines 278-284 on Page 14)**.

21. Line 269- 1.3 times as likely.

Response: Thank you for this. We have corrected **(Please see line 287 on Page 15)**.

22. Line 28- 1.2 times as likely.

Response: Thank you for this. We have corrected **(Please see line 296 on Page 15).**

23. Line 288- Also, prenatal breastfeeding intention is important as well.

Response: Thank you for this suggestion. We very much agree and we have also added this variable for future consideration **(Please see line 309 on Page 16)**.

I wish you great success in your continued efforts to improve care for mother-baby dyads.

Response: We also thank you for the detailed and through review of our manuscript. The comments and suggestions greatly improved our manuscript.

Responses to Reviewer 3 comments

Reviewer #3: Thank you for the team for their work in producing this manuscript. While I think it's an important question to understand determinants of SSC and that MICS data is high quality data to utilize for this analysis, I do have some questions or concerns regarding the analytical methodology and rationale behind some considerations.

Response: Thank you for the detailed review and thoughtful feedback.

1. Primarily, since this utilizes complex survey data, were appropriate weights applied when running the analyses to consider the survey design? With multi-level survey data, descriptive statistical findings will be incorrect without weighting. It was not clear in the methods whether this was done.

Response: Thank you for this comment. We have added these details **(Please see lines 153-155 on Page 7)**.

2. In the methods, it should also be clarified which variables were included in the multivariate analysis (was it all of the variables?) This can help a reader contextualize the findings in understanding what you controlled for. Also, the alpha level should be clarified.

Response: Thank you for this suggestion. We have described that we included those explanatory variables which provided a p-value <0.25 in the univariate regression analysis **(Please see lines 148-152 on Page 7)**.

3. For the SSC outcome, were there any 'don't know' responses? How were those coded? This is relevant for an outcome susceptible to recall bias.

Response: Thank you for this comment. Yes, the question on SSC includes 'Don't know' with 131 responses (over 1% of 11,992) and they were coded as zero for SSC (yes for the outcome and zero otherwise). This is a limitation of the study, and we have mentioned it in the limitations **(Please see lines 303-305 on Page 15)**.

4. What was the rationale behind including only ever-married women? What percentage of mothers are ever-married, and if it is a non-negligible proportion of mothers, might they be qualitatively different from never-married women?

Response: Thank you for this comment. Out of 44,341 women, 15,778 were never-married women and they were excluded from the study. In Afghanistan, a woman who is not married is not supposed to give birth due to cultural and religious value. In case there would cases of pregnancy among never-married women, it is not possible the women would seek any maternal health care. This means that their exclusion has no effect on our results, because they are not giving birth to probably practice SSC.

5. I agree that you included several key variables in your analysis, but I would have also included skilled birth attendance, which can greatly affect the quality of care of home births. You note this in the introduction, and also note that a substantial proportion of births occurred at home. In addition, you should include more detail or references on the literature review that led to the selection of variables.

Response: Thank you for this very useful suggestion. In the revised analysis, we have added the type of delivery provider, including deliveries conducted by relatives/other (this is mainly for those with deliveries at home) **(Please see Tables 1 and 2).** We have also added relevant citation for the inclusion of variables in the study **(Please see line 129 on 6).**

6. In the first paragraph of the results section, you should focus on the findings relevant to the outcome of SSC. You write about the characteristics of the whole population, but the relevant text here would be comparisons for how these characteristics differ between those with and without SSC.

Response: Thank you for this suggestion. We have revised it according to your advice **(Please see line 164 on Page 7)**.

7. I understand the importance of the data in Figure 2, but I am not sure that it is necessary, as it repeats the findings in Table 1.

Response: Thank you for this suggestion. We agree that this was duplicate. Hence, we have deleted figure 2.

8. For Table 2, since you don't discuss the findings for the univariate models, I would not include those here (perhaps in an appendix). Since the multilevel analysis implies that controlling for other variables are important, it implies that crude estimates are insufficient.

Response: Thank you for this suggestion. It is a conventional practice to provide crude ORs and adjusted ORs in one table so that the effect of confounders and modifiers on the ORs would be comparable. Thus, we request to keep them in Table 2.

9. In line 171-172, the findings should not only compare two wealth quintiles (which is a somewhat arbitrary division of the population), but rather speak more to the relationship between wealth and the outcome, perhaps including findings from the other wealth quintiles.

Response: Thank you. According to the MICS report, the wealth quintile has been created, using the household asset list and amenities and applying principal component analysis (so it is an arbitrary division of wealth status). The revised multivariate results show that there is no effect of wealth status on the outcome. Therefore, there shouldn't be any concern on the results related to wealth status **(Please see Table 2).**

10. On line 85, I think ‘emphasize’ should be ‘emphasizing’. The discussion and introduction were both well written.

Response: Thank you for your meticulous observation. We have corrected the term **(Please see line 84 on Page 4)**.

I hope these comments provide useful guidance.

Response: Thank you so much. The comments were very useful and helpful in improving the scientific rigor of the manuscript.

At last, we would like to thank once again the respected editor and esteemed reviewers for their constructive feedback and help, and we hope that the revised version of our manuscript will be deemed suitable for publication in this prestigious journal.

Thank you.

Sincerely,
Dr. Muhammad Haroon Stanikzai
Corresponding author
