# Supplementary material for: Prevalence and factors associated with mother and newborn skin-to-skin contact in Afghanistan
Source: PLoS One. 2025 May 21;20(5):e0324758. doi: 10.1371/journal.pone.0324758 (PMC12094735; doi:10.1371/journal.pone.0324758)
Supplement: Attachment — Submitted filename: Response_Letter_auresp_2.pdf [file pone.0324758.s004.pdf]

Dear Respected Editor,

We would like to thank you and the esteemed reviewers for the thoughtful evaluation of our manuscript (ID Number, **PONE-D-24-41027**) entitled ***"Prevalence and factors associated with mother and newborn skin-to-skin contact in Afghanistan"***. Please find our revised manuscript (with highlighted changes), which we believe is substantially strengthened now that we have incorporated reviewers' recommendations.

Response to Reviewers' comments

Responses to Reviewer 1 comments

Reviewer #2: Thank you for taking into consideration the suggested changes from my review as well as the other reviewers.

Response: We thank you and other reviewers for the thoughtful comments and review.

1. I noted that "access to radio" had a p-value of >0.25. Was this an exception to what you had stated in line 152 about the variables that included in your adjusted analysis (i.e. on lines 140-141, "access to media" was a combination of access to TV, radio and newspaper).

Response: Thank you so much for your meticulous observation. We agree with you that access to radio with a p-value of >0.25 shouldn't be included in the multivariate regression analysis, and this is why we created a combined variable "access to media" which includes access either to radio, TV, and newspaper. We revised this in Table1 to be consistent with our approach in Table2 **(Please see Table 1 on Pages 9 and 10)**. Furthermore, access to radio as a means of people's access to media is becoming more important, considering the increasing pressure of the Taliban's government on banning visual media and the deterioration of socioeconomical status in the country which may lead to the reliance of people more on radio as a means of access to media, particularly in rural areas of Afghanistan.

2. An important implication of your study findings is that TBA/ CHWs and relatives/others should receive education regarding the importance of SSC and how to do this safely. Not only does SSC help with bonding, but SSC can help with thermoregulation, prevent hypoglycemia, and stabilize respiratory effort among many other benefits. These are very important particularly in rural and remote areas, as you mentioned. I would suggest extending lines to 265-266. In addition to education for healthcare workers, public awareness campaigns may help to educate TBA/CHWs and relatives/others about the importance of SSC which may contribute to consistent SSC practices in Afghanistan.

Response: Thank you for this suggestion. We have added the following sentences **(Please see lines 267-269 on Page 14).**

3. Re: lines 232-233. Could your findings of a low rate of SSC be related to how you defined SSC? On lines 124-128 you indicated that to be coded a “yes” for SSC, mothers must have said yes to 1) baby was placed on bare chest after delivery and 2) breastfeeding initiated within the first hour of birth. Might this explain the difference in prevalence? Do other researchers using the MICS survey data combine those two actions? To me, they are separate. All babies benefit from SSC. For example, a mother may wish to bottle feed her baby, yet the baby would still benefit from SSC.

Response: Thank you so much. We agree with you that SSC was defined as placing the naked newborn on the mother’s bare chest on some previous studies. However, considering the importance of SSC within the first hour of childbirth is to promote initiation of early breastfeeding, we included both aspects in the definition and creation of the outcome “SSC” in our study. This is particularly important in the context of Afghanistan, where the way we defined SSC provides a more meaningful and practical aspect of maternal and child healthcare. Furthermore, the WHO has emphasized on the relevance of SSC and initiation of breastfeeding within 60 minutes of childbirth [1], and we followed the WHO’s recommendations on this. We have added the above justification in our paper **(please see lines 68-72, and 231-234).**

1. *WHO and UNICEF. Protecting, promoting and supporting breastfeeding in facilities providing maternity and newborn services: implementing the revised Baby-friendly Hospital Initiative 2018. ISBN 978-92-4-151380-7.*

I wish you great success in sharing these findings and improving care for women and babies in Afghanistan.

Response: We also thank you for the detailed and through review of our manuscript. The comments and suggestions greatly improved our manuscript.

At last, we would like to thank once again the respected editor and esteemed reviewers for their constructive feedback and help, and we hope that the revised version of our manuscript will be deemed suitable for publication in this prestigious journal.

Thank you.

Sincerely,

Dr. Muhammad Haroon Stanikzai
Corresponding author
